# Perfluoroalkyl substances and likelihood of stroke in persons with and without diabetes

**DOI:** 10.1177/1479164119892223

**Published:** 2019-12-16

**Authors:** Robert Hutcheson, Kim Innes, Baqiyyah Conway

**Affiliations:** 1 Department of Epidemiology, West Virginia University, Morgantown, WV, USA; 2 Department of Community Health, The University of Texas Health Science Center at Tyler, Tyler, TX, USA

**Keywords:** Perfluoroalkyl substances, diabetes, stroke, environmental contaminants

## Abstract

**Objective:**

The main objective of this study is to evaluate the relationship of perfluoroalkyl substances with stroke and any modifying influence of diabetes.

**Methods:**

Data on 3921 adults aged ⩾20 years with and 44,285 without diabetes were drawn from the C8 Health Project. Four perfluoroalkyl substances were investigated: perfluorohexane sulphate, C8 – perfluorooctanoic acid, perfluoroctane sulfonate and perfluorononaoic acid.

**Results:**

There were 238 cases of stroke among those with and 643 among those without diabetes. In analyses controlled for age, sex, race, diabetes duration, body mass index, high-density lipoprotein cholesterol, low-density lipoprotein cholesterol, C-reactive protein, kidney function and a history of smoking, a history of stroke was significantly inversely associated with serum perfluorohexane sulphate (odds ratio = 0.75, 0.64–0.88) and perfluoroctane sulfonate (odds ratio = 0.81, 0.70–0.90), but not perfluorooctanoic acid (odds ratio = 1.04, 0.94–1.15) or perfluorononaoic acid (odds ratio = 0.89, 0.70–1.14) among those with diabetes. Perfluoroalkyl substances demonstrated no association with stroke among those without diabetes (*p* interaction = 0.006 and 0.01 for perfluorohexane sulphate and perfluorooctanoic acid, respectively).

**Conclusion:**

In this large cross-sectional study, serum levels of perfluorohexane sulphate and perfluoroctane sulfonate were inversely associated with stroke among those with diabetes. Although mechanisms and implications for this diabetes-specific inverse relationship need to be further explored, our data suggest that perfluoroalkyl substances do not increase risk of stroke among persons with or without diabetes.

## Introduction

Perfluoroalkyl substances, or PFAS, are a class of highly fluorinated chemicals that have a wide range of uses and functional groups. PFAS are used in manufacturing many products that feature waterproofing, lubricating, non-stick and fire-suppression properties. They are absorbed in the ground from water and can also be absorbed by humans through drinking and contact with contaminated sources such as carpet, clothing, leather products, paper and packaging, coating additives, cleaning agents and firefighting foams.^1–3^ Because PFAS are persistent in the environment and are known to bio-accumulate in humans, the potential health effects of these compounds have received considerable attention.^4^

Notably, PFAS have been linked to a number of adverse health effects,^4,5^ including increased risk for various cancers and birth defects.^6,7^ In previous studies looking at risk factors for vascular diseases, PFAS have both been linked with higher cholesterol levels^8,9^ as well as dyslipidemia.^10^ High concentrations of PFAS have been both positively and inversely linked with diabetes, with both significant and non-significant associations observed for the inverse relationships.^11–14^ Mixed results have been observed for the relationship of PFAS with ischemic heart disease, with null^8,15^ to inverse relationships observed.^16^ For cerebrovascular disease, specifically stroke, two studies have examined the relationship of the PFAS perfluorooctanoic acid (PFOA) in worker populations, with one study observing an apparent protective relationship for stroke mortality^17^ and the other suggesting a positive relationship.^18^ A third study examined the relationship of PFOA with stroke in a combined population-based and worker cohort and also found mixed results, with prospective data suggesting a non-significant inverse relationship, but retrospective data suggesting a positive relationship with stroke incidence.^19^ Thus, the relationship of PFAS to stroke, an event that accounts for one out of every 20 deaths in the United States each year,^20^ remains unclear. Moreover, the association of PFAS other than PFOA with stroke has been little explored, and no studies have yet examined the potential modifying effect of diabetes, a major risk factor for stroke.^21,22^

We have recently observed inverse relationships between PFAS and coronary heart disease that was more pronounced among those with diabetes^16^ and between PFAS and kidney function and chronic kidney disease that was significantly stronger in those with diabetes.^23^ Using the C8 Health Project population, we examined the relationship of serum PFAS with stroke and the potential modifying influence of diabetes in a large sample of Appalachian adults, a population with among the highest rates of both diabetes and stroke in the United States.^20,24^

## Methods

The C8 Health Project^25,26^ was started as part of a legal settlement after PFOA (C8) had been found in the drinking water of residents of the mid-Ohio Valley in West Virginia and Ohio.^27^ Starting in August 2005 and ending in August 2006, baseline data were gathered on 69,030 people living and working in the six water districts, whose drinking water supplies had been contaminated with PFOA. As part of the project, blood samples were collected, and information was gathered on demographics, lifestyle characteristics, medical history, height, weight and other factors. Details on the project including consent, enrolment, data collection and reporting have been published^26^ and are reported online at (http://www.hsc.wvu.edu/resoff/research/c8/). In 2008, West Virginia University was granted access to the de-identified data from the C8 Health Project by Brookmar Inc, the organization in charge of the C8 Health Project. The study was approved by the West Virginia University Institutional Review Board.

Participation rates among eligible adults residing in the six affected water districts included in the C8 Health Project was 81%.^25^ For this current study, eligible participants included those at least 20 years of age at the time of clinical assessment, resulting in 54,099 eligible adults. A history of diabetes and a history of stroke were based on self-report of a physician diagnosis. Of the 54,457 adult participants, 5270 self-reported a physician diagnosis of diabetes and 1075 self-reported a history of stroke. Exclusion of participants with missing data on PFAS, stroke, diabetes and covariate data yielded a final analytic sample size of 48,206, including 3921 cases of diabetes and 881 cases of stroke.

Assay methods, blood processing and quality assurance measures have been previously described.^25,26,28^ In short, blood samples were taken from each participant; red blood cells and serum were immediately separated and refrigerated at the time of collection and were then transported to a laboratory for analyses on dry ice. PFAS assays were conducted using a protein precipitation extraction method which employed reverse-phase high-performance liquid chromatography and tandem mass spectrometry. A triple-quadrupole mass spectrometer in pre-selected reaction monitoring mode, monitoring for the M/Z transitions of PFAS species with an internal ^13^C PFAS standard corresponding to the target compound, was utilized for detection of each PFAS. Of the PFAS that were tested, four PFAS, including PFOA, PFOS (perfluoroctane sulfonate), PFHxS (perfluorohexane sulphate) and PFNA (perfluorononaoic acid), were found in over 97% of serum samples and were thus selected for investigation in this study. For these compounds, serum values that fell under the limit of detection were set at 0.25 ng/mL.

Estimated glomerular filtration rate was calculated based on the Chronic Kidney Disease – Epidemiology Collaboration (CKD-EPI) formula.^29^ Chronic kidney disease was defined as an estimated glomerular filtration rate (eGFR) of at least 60 mL/min/1.73 m^2^.

Univariate continuous data were analysed using the *t*-test or general linear models, while categorical data were analysed using the chi-square test. Logistic regression was used to determine the multivariable adjusted independent associations of serum PFAS levels to stroke. Multivariable models included race, sex, diabetes duration, body mass index (BMI), high-density lipoprotein (HDL) cholesterol, low-density lipoprotein (LDL) cholesterol, C-reactive protein, eGFR (mL/min/1.73 m^2^) and a history of smoking. To evaluate the potential modifying effect of diabetes on the association between each of the four PFAS and a history of stroke, we conducted multivariate analyses stratified by diabetes status. The interaction between diabetes and each PFAS was subsequently tested by including the respective multiplicative interaction term in the adjusted statistical model in the full population of those with and without diabetes. Data were analysed using SAS, version 9.4 (Cary, North Carolina).

## Results

Characteristics of the study population stratified by diabetes status are presented in Table 1. Persons with diabetes tended to be older and non-white. They tended to have a higher BMI but lower cholesterol levels and kidney function. They were also more likely to have a history of stroke, chronic kidney disease and a history of smoking. PFOA, PFOS and PFHxS concentrations were significantly higher in those with versus without diabetes. PFNA concentrations were similar in the two groups.

**Table 1.** Characteristic of C8 Health Project adults age ⩾ 20 years of age, stratified by diabetes status.

	Diabetes *N* = 3921	No diabetes *N* = 44,285	*p* value
Age, years	58.0 ± 13.6	45.0 ± 15.6	<0.0001
Sex, female	52.0 (2038)	54.2 (24,010)	0.007
Race, white	98.2 (3850)	98.9 (43,799)	<0.0001
Diabetes duration, years	6.5 (3.2–13.3)	–	–
BMI, m/kg^2^	33.0 ± 9.5	28.2 ± 7.4	<0.0001
HDLc, mg/dL	46.8 ± 12.2	50.1 ± 14.5	<0.0001
LDLc, mg/dL	98.6 ± 36.5	114.0 ± 34.7	<0.0001
C-reactive protein, mg/dL^a^	2.6 (1.1–6.0)	1.8 (0.80–4.2)	<0.0001
eGFR, mL/min/1.73 m^a,b^	77.1 ± 22.1	88.0 ± 19.0	<0.0001
Chronic kidney disease	21.6 (848)	7.2 (3171)	<0.0001
A history of stroke/TIA	6.1 (238)	1.5 (643)	<0.0001
A history of smoking	55.1 (2161)	52.3 (23,174)	<0.0001
Perfluroakly acids	–	–	–
PFHxS^a^	2.8 (1.8–4.3)	3.0 (1.9–4.8)	<0.0001
PFOA^a^	28.7 (12.9–73.6)	27.6 (13.4–70.4)	0.97
PFOS^a^	21.4 (13.8–31.9)	20.1 (13.5–29.0)	0.01
PFNA^a^	1.3 (1.0–1.8)	1.4 (1.0–1.8)	<0.0001

Data are presented as means ± SD, median (IQR) or percent (*n*).

BMI: body mass index; HDLc: high-density lipoprotein cholesterol; LDLc: low-density lipoprotein cholesterol; TIA: transient ischaemic attack; PFHxS: perfluorohexane sulphate; PFOA: perfluorooctanoic acid; PFOS: perfluoroctane sulfonate; PFNA: perfluorononaoic acid.

a Natural logarithmically transformed before analyses.

b CKD-EPI formula.

**Figure 1.**
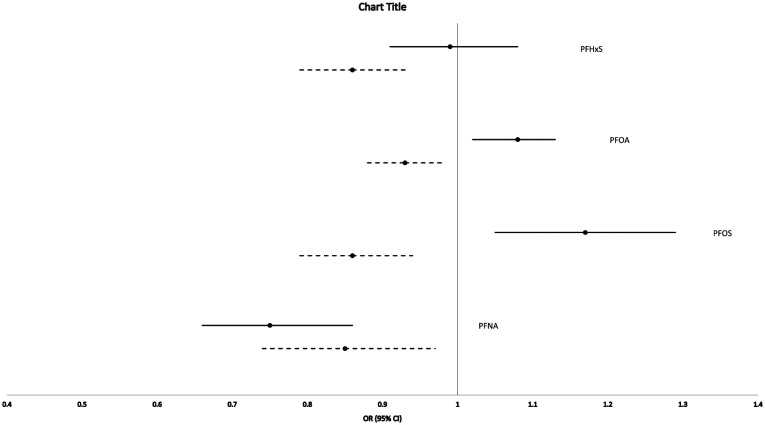
Univariate and age-adjusted association of PFAS with stroke in the C8 Health Population. Solid lines represent univariate analyses. Dashed lines represent age-adjusted analyses. PFHxS: univariate OR = 0.99 (0.91–1.08), age-adjusted OR = 0.86 (0.79–0.93); PFOA: univariate OR = 1.08 (1.02–1.13), age-adjusted OR = 0.93 (0.88–0.98); PFOS: univariate OR = 1.17 (1.05–1.29), age-adjusted OR = 0.86 (0.79–0.94); PFNA: univariate OR = 0.75 (0.66–0.86), age-adjusted OR = 0.85 (0.74–0.97).

A history of stroke was reported in 1.8% (*n* = 881) of the population as a whole, in 6.1% (*n* = 238) of those with diabetes and 1.5% (*n* = 643) of those without diabetes. As older individuals would have a greater prevalence of stroke and would also have a higher lifetime exposure to PFAS,^15^ we thus first tested for confounding by age in the association of PFAS with stroke. Figure 1 depicts the unadjusted and age-adjusted association of each PFAS with stroke. These data suggest substantial confounding by age as the relationship between PFAS and stroke generally became stronger, and for two of the PFAS (PFOA and PFOS), the relationship went from being positively associated to negatively associated with stroke. Hereafter, all analyses were age adjusted in our base models.

Table 2 presents the multivariable adjusted association of stroke with the four PFAS. As also depicted in Figure 1, in analyses adjusted only for age, all four PFAS were significantly and inversely associated with stroke, with odds ratios (ORs) ranging from 0.85 (0.74–0.97) for PFNA to 0.93 (0.88–0.98) for PFOA. Additional adjustment for sex, diabetes duration, BMI, HDL cholesterol, LDL cholesterol, C-reactive protein, eGFR and a history of smoking modestly attenuated these inverse associations, with only those of PFHxS [OR = 90, 95% confidence interval (CI) = 0.83–0.98] and PFOS (OR = 0.90, 95% CI = 0.82–0.98) remaining significantly associated with stroke.

**Table 2.** Association of the PFAS with stroke in the C8 Health Population.

	Model 1		Model 2	
	OR (95% CI)	*p* value	OR (95% CI)	*p* value
PFHxS^a^	0.86 (0.79–0.93)	0.0003	0.90 (0.83–0.98)	0.02
PFOA^a^	0.93 (0.88–0.98)	0.01	0.96 (0.91–1.01)	0.12
PFOS^a^	0.86 (0.79–0.94)	0.0009	0.90 (0.82–0.98)	0.02
PFNA^a^	0.85 (0.74–0.97)	0.02	0.90 (0.79–1.02)	0.10

PFAS: perfluoroalkyl substances; OR: odds ratio; CI: confidence interval; PFHxS: perfluorohexane sulphate; PFOA: perfluorooctanoic acid; PFOS: perfluoroctane sulfonate; PFNA: perfluorononaoic acid.

Model 1: Adjusted for age.

Model 2: Adjusted for age, sex, race, diabetes duration^a^, BMI, HDL cholesterol, LDL cholesterol, C-reactive protein^a^, eGFR and a history of smoking.

a Naturally logarithmically transformedd before analyses.

Table 3 depicts the relationship of the four PFAS with stroke stratified, by diabetes status. Significant effect modification by diabetes status with stroke was observed for the sulphur-containing PFAS. PFOS (OR = 0.81, 95% CI = 0.70–0.90) and PFHxS (OR = 0.75, 95% CI = 0.64–0.88) both showed significant, inverse relationships with stroke among those with diabetes but no relationship among those without diabetes (*p* interaction = 0.006 for PFOS and 0.01 for PFHxS). Conversely, PFOA was inversely associated with stroke among those without diabetes (OR = 0.94, 95% CI = 0.88–1.00) but not among those with diabetes (OR = 1.04, 95% CI = 0.94–1.15), although the interaction was non-significant (*p* interaction = 0.69).

**Table 3.** Association of PFAS with stroke, stratified by diabetes status, in the C8 health population.

Model 1				
	Diabetes		No diabetes	
	OR (95% CI)	*p* value	OR (95% CI)	*p* value
PFHxS^a^	0.70 (0.60–0.82)	<0.0001	0.97 (0.88–1.08)	0.61
PFOA^a^	1.00 (0.90–1.11)	0.98	0.92 (0.87–0.98)	0.01
PFOS^a^	0.76 (0.66–0.88)	0.0002	0.94 (0.84–1.06)	0.31
PFNA^a^	0.81 (0.63–1.03)	0.08	0.88 (0.75–1.03)	0.10
Model 2				
	Diabetes		No diabetes	
	OR (95% CI)	*p* value	OR (95% CI)	*p* value
PFHxS^a^	0.75 (0.64–0.88)	0.0004	0.99 (0.90–1.10)	0.91
*p* interaction^b^	0.006			
PFOA^a^	1.04 (0.94–1.15)	0.47	0.94 (0.88–1.00)	0.04
*p* interaction^b^	0.69			
PFOS^a^	0.81 (0.70–0.90)	0.004	0.97 (0.86–1.08)	0.54
*p* interaction^b^	0.01			
PFNA^a^	0.89 (0.70–1.14)	0.37	0.91 (0.78–1.06)	0.22
*p* interaction^b^	0.83			

PFAS: perfluoroalkyl substances; OR: odds ratio; CI: confidence interval; PFHxS: perfluorohexane sulphate; PFOA: perfluorooctanoic acid; PFOS: perfluoroctane sulfonate; PFNA: perfluorononaoic acid.

Model 1: Adjusted for age.

Model 2: Adjusted for age, sex, race, diabetes duration^a^, BMI, HDL cholesterol, LDL cholesterol, C-reactive protein^a^, eGFR and a history of smoking.

a Naturally logarithmically transformedd before analyses.

b *p* value for interaction between the specific PFAS and diabetes status in Model 2.

## Discussion

In this study of nearly 50,000 Appalachian adults, we investigated the association of serum levels of four PFAS with stroke and evaluated the potential modifying influence of diabetes on these associations. We found that higher levels of each of the sulphur-containing PFAS were associated with a lower likelihood of stroke. Stratifying by diabetes status generally yielded similar inverse associations, though mainly significant only among those with diabetes. To our knowledge, this is the first study to investigate the relationship of PFAS with stroke among persons with diabetes.

The few studies that have investigated the relationship of PFAS with stroke have been restricted to PFOA and largely to worker populations. Leonard and colleagues found a decreased risk of stroke death among workers at the Parkersburg, West Virginia DuPont polymer plant, a population of workers exposed to high levels of PFOA, compared to both the US general population and the West Virginia general population. They also observed non-significant decreased risk compared to a DuPont worker population composed of eight states in the area surrounding West Virginia.^17^ By contrast, Lundin et al.^18^ found an increased risk of stroke death associated with PFOA exposure among a worker population at the 3M Company in Cottage Grove, MN. In an study composed of approximately two-thirds of the population reported on in our analyses, that is, the adult C8 Health Project population, plus an additional worker population of the Parkersburg, WV DuPont plant, Simpson et al.^19^ observed inconclusive relationships between PFOA exposure and stroke risk. In their analyses, PFOA exhibited a modest positive association with stroke risk when examined retrospectively but a non-significant inverse relationship when examined prospectively. In their analyses, diabetes was not examined specifically. Our study, by stratifying by diabetes status and examining additional PFAS, expands upon their findings. In agreement with Simpson et al.’s prospective analyses, we observed a weak and non-significantly inverse relationship between PFAS and stroke in the population as whole after adjustment for potential confounders. We also observed this non-significant relationship in the population with diabetes for PFOA and PFNA. However, for the sulphur-containing PFAS, not examined in the Simpson et al. study,^19^ we observed significant inverse associations in the diabetic population, while no relationship was observed in the non-diabetic population.

While underlying mechanisms remain speculative, pathways by which PFAS may decrease stroke risk are several. These include the potential reduction in BMI in those exposed to PFAS suggested in some,^15,30,31^ but not all studies;^32^ the high oxygen-carrying capacity of certain PFAS indicated in several prior studies;^33–36^ and their insulin-sensitizing and anti-inflammatory properties.^37–39^ PFAS have also been shown to reduce vascular hypoxia, a trigger for vascular disease and atherosclerosis.^33,34^ In addition, PFAS has been inversely associated with hypertension,^15^ an established risk factor for stroke.

PFAS are synthetic ‘hydrocarbon’ compounds in which the fluorine either partially or completely replaces the hydrogen atoms. This chemical structure gives PFAS both oleophobic and hydrophobic characteristics. PFAS are persistent environmental contaminants due to their strong carbon-fluorine bonds which resist environmental degradation. However, the fluorine replacement of carbon in PFAS makes PFAS very good oxygen carriers with an oxygen solubility in perfluorocarbons that is 25 times higher than haemoglobin.^33,34^ They are also able to load and unload oxygen at twice the rate of haemoglobin, making them better oxygen transporters. Thus, PFAS could potentially decrease the risk for stroke occurrence by limiting hypoxia-induced inflammation by reducing the oxidative stress caused by hypoxia. While we cannot explain the apparent stronger relationships of the sulphur-containing PFAS with stroke, or why this was more pronounced in those with diabetes, it is possible that PFHxS and PFOS exert a more potent anti-hypoxic effect due to their longer half-lives.^40,41^ This may be particularly beneficial among persons with diabetes, a condition often characterized by generalized hypoxia.^42^ However, no studies have yet determined if the common industrial PFAS included in this study are oxygen carriers or can be used to deliver oxygen to cells.

Strengths of our study include the large sample size and high participation rate. Our population of Appalachian adults included nearly 48,000 adults, and the participation rate among eligible adults exceeded 80%.^25^ In addition, information was available on a wide array of biomarkers, as well as on multiple potential confounders.

Our study also had several limitations. Since this was a cross-sectional study, no conclusions about causality can be drawn. This population also comprised a predominately white, Appalachian population, limiting generalizability to other racial and ethnic groups. Another limitation is that diabetes and stroke status were determined via-self report of a physician diagnosis; thus, misclassification of both diabetes and stroke status is possible. However, any misclassification was likely due to under-ascertainment of diabetes or stroke, which would have resulted in biasing our results towards the null, that is, our observed results being an underestimation of the relationship between PFAS and stroke. Survival bias, in which participants with especially high or low levels of PFAS could have died before the start of the study, must also be considered, as the drinking water had been contaminated for more than 50 years. Thus, sicker people with high serum PFAS levels resulting in stroke, particularly those with both diabetes and stroke, may have already died before entering the study, and thus a harmful prospective relationship between PFAS and stroke is not being observed. Our findings comparing crude analyses with age-adjusted analyses, in which older individuals would most likely have had a longer lifetime exposure to PFAS, suggest that survival bias is not a strong explanation for our results, since the relationships became more strongly inverse upon adjustment for age.

In light of both the limitations of our study and the potential protective relationship between certain PFAS and stroke risk among persons with diabetes, rigorous prospective research among persons with diabetes is clearly warranted to confirm our findings. If our findings are confirmed, future studies should investigate mechanistic pathways in which PFAS may protect against stroke. These studies may be animal models evaluating the degree to which PFAS protect against hypoxia, as well as whether the PFAS examined in our study can serve as an oxygen-carrying replacement for haemoglobin, function as an anti-glycaemic agent (by stimulating insulin production or by serving as an insulin sensitizer) and/or whether the potential protective property of PFAS is through other mechanisms. Moreover, human studies could examine whether the association of PFAS with stroke reduction is primarily through prevention of hemorrhagic versus ischemic stroke; if the putative protective effects of PFAS are predominantly related to ischemic stroke prevention, this would provide further evidence of PFAS acting via hypoxia inhibition.

## Conclusion

In conclusion, in this large cross-sectional study of nearly 50,000 adults, serum levels of PFHxS and PFOS were significantly and inversely associated with stroke; these associations were significantly more pronounced among those with diabetes. Mechanisms and implications for this diabetes-specific inverse relationship need to be further explored. While our results should not be interpreted as suggesting that exposure to PFAS is beneficial, our data do suggest that PFAS do not increase the risk of stroke among persons with or without diabetes. Moreover, PFAS may offer protection from stroke in individuals with diabetes, and this is an area that warrants further investigation
